# Novel Enzymes From the Red Sea Brine Pools: Current State and Potential

**DOI:** 10.3389/fmicb.2021.732856

**Published:** 2021-10-27

**Authors:** Dominik Renn, Lera Shepard, Alexandra Vancea, Ram Karan, Stefan T. Arold, Magnus Rueping

**Affiliations:** ^1^KAUST Catalysis Center (KCC), Division of Physical Sciences and Engineering, King Abdullah University of Science and Technology, Thuwal, Saudi Arabia; ^2^Institute of Organic Chemistry, RWTH Aachen, Aachen, Germany; ^3^Computational Bioscience Research Center (CBRC), Division of Biological and Environmental Science and Engineering, King Abdullah University of Science and Technology, Thuwal, Saudi Arabia; ^4^Centre de Biologie Structurale, CNRS, INSERM, Université de Montpellier, Montpellier, France; ^5^Institute for Experimental Molecular Imaging (ExMI), University Clinic, RWTH Aachen, Aachen, Germany

**Keywords:** extremozymes, biocatalysis, Red Sea, brine pools, extremophile

## Abstract

The Red Sea is a marine environment with unique chemical characteristics and physical topographies. Among the various habitats offered by the Red Sea, the deep-sea brine pools are the most extreme in terms of salinity, temperature and metal contents. Nonetheless, the brine pools host rich polyextremophilic bacterial and archaeal communities. These microbial communities are promising sources for various classes of enzymes adapted to harsh environments – extremozymes. Extremozymes are emerging as novel biocatalysts for biotechnological applications due to their ability to perform catalytic reactions under harsh biophysical conditions, such as those used in many industrial processes. In this review, we provide an overview of the extremozymes from different Red Sea brine pools and discuss the overall biotechnological potential of the Red Sea proteome.

## Introduction

Over the past decade, the interest and demand for green chemistry and green biotechnology has increased steadily (Wenda et al., 2011). Consequently, the search for enzymes that can be employed in more sustainable and thus overall ‘greener’ industrial processes has intensified (Li et al., 2012; Singh et al., 2016; Chapman et al., 2018). According to a Business Communications Company (BCC) research report, the global market for industrial enzymes is estimated to reach US $7 billion by 2023, with an expected annual growth rate of 5% (Shahani, 2018).

Numerous enzyme classes with potential industrial applications have been identified (Honda, 2017; Basso and Serban, 2019). The majority of these classes are already used in a few very specific industrial applications (Robic et al., 2017). In particular, hydrolases, peptidases, lipases, cellulases, and amylases are highly used (Shahani, 2018). Nonetheless, the commercial enzyme market struggles to respond to the increasing demands of the biotechnology sectors. The main obstacle is that most enzymes cannot survive harsh industrial conditions and are not suitable for iterative biocatalysis cycles. Thus, enhancing enzyme stability can markedly lower industry expenses (Jin et al., 2019). For some reaction conditions, enzymes can be sufficiently stabilized by surface modifications, e.g., PEGylation or immobilization (Dumorne et al., 2017; Ellis, 2019). However, ideally, industry would need enzymes that can naturally and reproducibly withstand a combination of several extreme conditions (pH, temperature, salinity, organic solvents, and/or aerification) (Sarmiento et al., 2015; Dumorne et al., 2017; Kara and Liese, 2019). Naturally, halotolerant enzymes are of particular interest for biotech applications because high salt concentrations correspond to low water activity and hence to increased tolerance to organic solvents.

A potential source for such polyextremozymes is the proteomes of microorganisms that thrive under environmental conditions which were previously considered unhabitable (Hough and Danson, 1999; Dumorne et al., 2017). These organisms have developed cellular and molecular mechanisms to withstand multiple ecological extremes, including high or low temperatures, acidic or basic pH, high salinity, and/or high metal concentrations (Eichler, 2001). Enzymes from these polyextremophiles hold the promise of fulfilling industrial demands (Kara and Liese, 2019) because the conditions of their natural habitats are similar to those occurring in industrial processes (Niehaus et al., 1999; Eichler, 2001; van den Burg, 2003; Gomes and Steiner, 2004; Raddadi et al., 2015; Coker, 2016).

Generally, extremozymes can be classified according to habitat, e.g., as cold-tolerant (psychrophilic), temperature-tolerant (thermophilic and hyperthermophilic), acid-tolerant, alkali-tolerant, and salt-tolerant (halophilic) (Sarmiento et al., 2015). Every class of these enzymes has evolved specific structural and/or mechanistic adaptations (Feller, 2003; Collins and Margesin, 2019). Psychrophilic enzymes, for example, increase their catalytic activity at low temperatures through increased structural flexibility and greater exposure of hydrophobic residues compared to thermophilic or hyperthermophilic extremozymes (Szilágyi and Závodszky, 2000). However, the same features can lead to poor stability at higher temperatures. Thus, tolerance to one type of extreme condition can lead to weaknesses in another. Some extremozyme classes, such as cold-tolerant, acid-tolerant, alkali-tolerant, and salt-tolerant classes, are already employed in industrial applications (Gomes and Steiner, 2004; Coker, 2016). Nevertheless, discovering and characterizing enzymes with appropriate activity and stability under polyextremophilic conditions continues to be an essential aim in enzymology (Bruins et al., 2001; de Champdore et al., 2007; Suriya et al., 2016; Krüger et al., 2018).

The discovery of extremozymes has accelerated as a result of substantial progress in next-generation sequencing (NGS) technology (Buermans and den Dunnen, 2014). Current NGS technology enables metagenomics to be used as a routine technique in environmental microbiology (Bragg and Tyson, 2014; Alves et al., 2018).

Together, NGS, metagenomics, and metaproteomics (VerBerkmoes et al., 2009; Siggins et al., 2012; Wang et al., 2016; Speda et al., 2017) provide a powerful platform to investigate the microbial communities from remote and polyextreme habitats (Rekadwad et al., 2017).

In particular, the field of marine microbial ecology was boosted by NGS-metagenomic platform technology, showing the biodiversity of various marine environments and revealing previously unknown microbial communities (Kodzius and Gojobori, 2015; Coutinho et al., 2018). The large number of metagenomic datasets generated by those platforms has facilitated the identification of a huge number of metagenomes (Kennedy et al., 2010; Barone et al., 2014; Ziko et al., 2019). These microbial gene datasets helped to obtain a better understanding of adaptive mechanisms and community interactions. These data also allowed us to estimate the pharmaceutical and biotechnological impacts and the application areas of the discovered genes (Kennedy et al., 2010; Barone et al., 2014; Ziko et al., 2019).

The Red Sea has unique ecological factors and characteristics, especially high temperature even at its bottom (22°C), high salinity and high UV radiation (Rasul et al., 2015). Thus, the Red Sea is considered a ‘laboratory’ for studying life under a ‘global warming’ scenario (Bellworthy and Fine, 2018). Approximately 25 brine pools are located at the bottom of the Red Sea. Owing to the location, small size and (for some) relatively recent discovery of these brine pools, their microbial communities are among the least studied communities in marine environments (Thompson et al., 2013; Behzad et al., 2016; Ziko et al., 2019). These oligotrophic marine ecosystems display unique physiochemical properties and microbial communities (Behzad et al., 2016).

Herein, we focus on extracted, characterized (poly)extremozymes from Red Sea brine pools and the biotechnological potential of the Red Sea proteome. Furthermore, we discuss the limitations of biomining the Red Sea proteome. Several reviews already cover the population genetics and the microbiome of the Red Sea brine pools, or extremozymes from different sources (Barozzi et al., 2018; Jin et al., 2019; Sharma et al., 2019; Varrella et al., 2020).

## The Red Sea and Brine Pool Characteristics

### The Red Sea

The Red Sea is a seawater inlet of the Indian Ocean situated between Africa and the Arabian Peninsula. Located between an arid and semi-arid desert, the Red Sea has a length of approx. 2,000 km, with a maximum width of 355 km and a maximum depth of 3,039 m. These dimensions result in a surface area of approximately 4.6 km × 105 km and a sea water volume of approximately 2.5 × 10^5^ km^3^ of (salt) water (Rasul et al., 2015; Berumen et al., 2019).

The Red Sea is among the least explored marine environments (Rasul et al., 2015; Carvalho et al., 2019). It is also a very special ecological environment because (i) it is the northernmost tropical sea; (ii) it displays unique physical and chemical characteristics; and (iii) it exhibits substantial variation in extreme ecological niches.

The Red Sea is a marine environment with unusual physical and chemical parameters. It has high water temperatures all year and at all depths, with a minimum of 21°C. The Red Sea is also permanently exposed to strong UV radiation, has high salinity (140 and 255 ‰) (Schmidt et al., 2015; Berumen et al., 2019), and exhibits an unusually low average nutrient level. Regular dust storms (approximately 15–20 per year) (Jish Prakash et al., 2015) cool the Red Sea and affect the salinity distribution (Berumen et al., 2019). The few metagenomic studies conducted on the Red Sea revealed that the microbiota, and therefore the proteome, differ markedly from those found in other marine environments (Thompson et al., 2013; Abdallah et al., 2014). Of particular interest for biotechnological discovery of (poly)extremozymes are the Red Sea’s most extreme niches, the brine pools (Schmidt et al., 2015).

### The Brine Pools in the Red Sea

**FIGURE 1:**
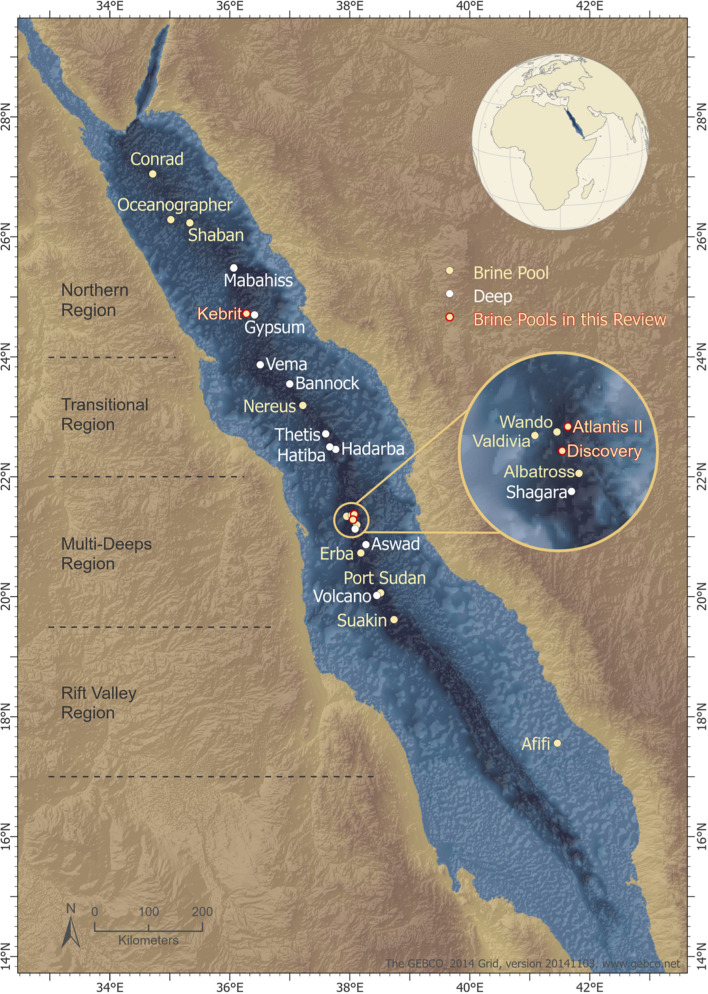
Location of the deeps along the Red Sea rift axis. The topographic depression is segmented by its geotectonic classification, (Bonatti, 1985) and the segments consist of (i) a northern region, (ii) a transitional region, (iii) a multi-deeps region, and (iv) a rift valley region. Deeps are marked in white; brine pools are marked in beige; brine pools from which enzymes were extracted are marked in red. The geotectonic classification of Red Sea segments (gray dotted lines) is adapted from Bonatti (1985). The map was created by Ute Langner, Red Sea Research Center KAUST.

The movement of the African and Arabian tectonic plates caused a topographic depression along the mid-axial rift valley within the Red Sea. This depression is approximately 1,500–2,800 m deep and is characterized by hypersalinity, acidity, and anoxicity (Rasul et al., 2015). Twenty-five deep-sea brine pools (Schmidt et al., 2015) (Figure 1) have been identified in this depression after half a century of research (Rasul et al., 2015; Carvalho et al., 2019).

These brine pools consist of highly dense brine-seawater layers and display increasing salinity, thus preventing their layers from merging with the surrounding seawater. Nonetheless, these brine-seawater interfaces allow the exchange of organic material from the overlaying water. This exchange enables carbon intake for the communities living within the brine pools. Nutrient access along the salinity gradient fluctuates greatly, leading to variations in the metabolic processes in the microorganisms inhabiting different layers (Antunes et al., 2011; Berumen et al., 2019).

Atlantis Deep II, Discovery Deep, and Kebrit are the best studied brine pools of the Red Sea (Hartmann et al., 1998; Swift et al., 2012; Schmidt et al., 2015). They are located below 1.5 km depth, classifying them as deep-sea brine pools (Antunes et al., 2011; Schmidt et al., 2015; Elbehery A. H. A. et al., 2017). They display polyextremophilic conditions including (i) high temperatures (up to 60°C or higher); (ii) high salinity (up to seven times higher than the surrounding deep sea water); (iii) low pH values; (iv) highly metalliferous deposits, including iron (Fe), manganese (Mn), zinc (Zn), nickel (Ni), copper (Cu), lead (Pb), cobalt (Co), barium (Ba), silicon (Si), and lithium (Li) in dissolved form, and more rarely silver (Ag) or gold (Au); (v) low dissolved oxygen concentrations (up to 40 times lower than in the “deep zone”); or (vi) completely anaerobic conditions (Gurvich, 2006; Antunes et al., 2011). However, the temperature, salinity, pH and composition of the metalliferous sediments vary between each of the known brine pools, (Antunes et al., 2011) leading to the presence of specific and unusual microbial communities.

With a volume of approximately 17 km^3^, Atlantis II Deep is the largest pool (Anschutz and Blanc, 1996) in the region. It is located at a depth of 1,900–2,200 m (Anschutz and Blanc, 1996) and is hydrothermally active. Atlantis II Deep is hot (approximately 68°C), hypersaline (up to 257 psu), acidic (pH of 5.3), and has high metal content (Backer and Schoell, 1972; Anschutz et al., 2000; Abdallah et al., 2014; Schmidt et al., 2015). The metals in Atlantis II Deep appear mostly as sulfides of Zn, Cu, Mn, Co, and Fe, with significant amounts of Ag and Au (Danielsson et al., 1980; Bertram et al., 2011).

Discovery Deep is located at 2,100 m depth, in close proximity to Atlantis II Deep. Both brine pools have subsurface connections and are geochemically and physically similar (Backer and Schoell, 1972; Hartmann et al., 1998; Schmidt et al., 2003; Antunes et al., 2011). However, the conditions in Discovery Deep are less extreme than those in Atlantis II Deep (Abdallah et al., 2014; Schmidt et al., 2015). Discovery Deep’s metal content consists mostly of Si, Mn, and Fe (Danielsson et al., 1980).

Kebrit Deep is 2.5 km^2^ in size, one of the smallest known Red Sea brine pools, located at 1,549 m depth. Kebrit Deep is not a hot brine, with temperatures of 21–23°C, but it contains several inactive vents at the rim. The salinity is approximately 242 psu, and the pH is approximately 5.2. The brine layer is 84 m thick, which allows anaerobic conditions, and the oxygen levels reach zero (Backer and Schoell, 1972; Hartmann et al., 1998; Eder et al., 2001; Schmidt et al., 2003). Kebrit Deep is rich in hydrogen sulfide (H_2_S), containing approximately 4–12 mg/L (Hartmann et al., 1998). Underneath the brine, sediments rich in heavy metals can be found (Zierenberg, 1990; Anschutz et al., 2000; Schmidt et al., 2015). These sediments mainly contain Fe, Zn, and Pb, and a small amount of Ni (Blum and Puchelt, 1991).

Several polyextremophilic microbes thrive under these conditions (Eder et al., 2001; Wang et al., 2011, 2013; Siam et al., 2012; Bougouffa et al., 2013; Abdallah et al., 2014; Grotzinger et al., 2014; Guan et al., 2015; Ziko et al., 2019). These microbial communities are adapted not only to high salinity (4–26%) but also to elevated temperatures, low oxygen concentrations, and high concentrations of heavy metals (Craig, 1966; Hartmann et al., 1998; Schmidt et al., 2003; Antunes et al., 2011). As a result of this polyextremophilic adaptation, the microbial enzymes are extremely stable and produce various natural compounds with potential industrial application (Ziko et al., 2019). Hence, they are promising candidates for improved biocatalysis in the industrial setting and may help industries switch to more sustainable and ecologically friendly alternatives.

## Mining of the Red Sea Enzyme Pool

The combination of adapted computational and experimental tools is necessary to harness the biotechnological potential of the Red Sea brine pools.

### Metagenomic Data and Database

The high inconsistency between culturable bacteria and expected bacterial count in environmental samples indicates that we currently know only a small fraction of the bacterial diversity in nature (Amann et al., 1995). The single-amplified genome (SAG) strategy (Alneberg et al., 2018) for retrieving genomes from samples without the need for cultivation in combination with high-throughput NGS technologies for culturable bacteria is a valuable approach for mining metagenomic data from the Red Sea and the Red Sea brine pools (Kamanda Ngugi et al., 2015). The speed of genome sequencing by NGS technologies is orders of magnitude higher than that of experimental testing, creating a ‘bottleneck’ in downstream experimental analysis (Médigue and Moszer, 2007; Pop and Salzberg, 2008; MacLean et al., 2009; Rekadwad et al., 2017). The annotation of these newly sequenced genomes relies primarily on computational methods (Siezen and van Hijum, 2010; Richardson and Watson, 2012) capable of extrapolating the enzyme function from available biological information, which is ideally derived from multiple sources (Poptsova and Gogarten, 2010). The initial annotation by information integration and/or combination can simplify and facilitate downstream analysis and experiments (Castro et al., 2005; Sansone et al., 2012). Nevertheless, this annotation is still prone to errors (Brenner, 1999; Poptsova and Gogarten, 2010; Klimke et al., 2011; Salzberg, 2019). The biocomputational field provides numerous tools for taxonomic and functional annotation of metagenomic data, which are all intended to overcome the problem of annotation errors and thus ease downstream experimental analysis by avoiding incorrectly annotated genes and gene functions.

There is a growing amount of genomic data available, mainly from Atlantis II, Discovery, and Kebrit, but also from the lesser-studied brine pools Nereus, Erba, and Shaban (Alam et al., 2013; Guan et al., 2015). However, to date, there is only one public database compiling the available Red Sea genomic data: the INtegrated Data Warehouse of MIcrobial GenOmes (INDIGO^1^). INDIGO aims to be a dedicated database of metagenomic information of microorganisms from the Red Sea. It contains fully annotated genomes of bacterial and archaeal species isolated from the Red Sea. INDIGO uses the Automatic Annotation of Microbial Genomes (AAMG) algorithm, which comprises different annotation methods, in combination with profile and pattern matching (PPM). This algorithm combination aims to reduce the level of uncertainty in gene annotation and therefore lowers the likelihood of false-positive enzyme function annotation (Grotzinger et al., 2014). Furthermore, INDIGO derives and combines information from several databases, such as the CDD (Conserved Domains Database) (Lu et al., 2020), GO (Gene Ontology) (Ashburner et al., 2000; Consortium T. G. O., 2020), InterPro (Hunter et al., 2009), KEGG (Kyoto Encyclopedia of Genes and Genomes) (Kanehisa and Goto, 2000), and UniProt (Consortium T. U., 2020), to provide as much information as possible (Alam et al., 2013).

Having such a database is necessary for a systematic screening of the Red Sea in search of extremophilic enzymes. However, a wider input and contribution network is necessary to keep the collection up-to-date and growing.

### Microbial Expression Systems

Genomic data, even if functionally annotated, need to be complemented by experimental testing to allow successful enzyme discovery for biotechnology. Most industrial applications rely on recombinantly produced microbial proteins. The choice of the most suitable expression system for the efficient production of the recombinant protein depends on several factors, such as the growth characteristics of the host cell, expression levels, intracellular or extracellular-segregation expression, post-translational modifications, and biological activity (Liu et al., 2013). However, the main parameters to consider are (i) the enzyme production rate and (ii) the yield of the expression system (Liu et al., 2013).

The most commonly used expression system is the heterologous host *Escherichia coli* (*E. coli*) because of the short doubling time and its arsenal of established expression protocols, cloning methods, and a broad range of available plasmid vectors. Proteins adapted to high temperature or harsh pH are generally well-expressed in *E. coli*. However, *E. coli* has proven to be poorly suited for the expression of some proteins from (poly)extremophilic microbes, in particular from halophiles (Grotzinger et al., 2018). To be active at low water and at high salt concentrations, halophilic proteins display multiple acidic amino acid residues, generating a negative surface charge and allowing their solubility in high salt (Danson and Hough, 1997; Mevarech et al., 2000; Siglioccolo et al., 2011; Sinha and Khare, 2014). This feature, however, promotes their misfolding and aggregation under conditions of low ionic strength, as prevailing in *E. coli* cells (Danson and Hough, 1997; Mevarech et al., 2000; Siglioccolo et al., 2011; Sinha and Khare, 2014; Grotzinger et al., 2018).

Nonetheless, expression protocols to produce and purify recombinant halophilic proteins from several haloarchaea in *E. coli* have been developed (Salin et al., 1988; Camacho et al., 2002; Esclapez et al., 2006; De Castro et al., 2008). However, recombinant proteins are usually obtained as inclusion bodies and are then refolded using slow or rapid dilution in a high salt concentration buffer to recover the expressed protein (Connaris et al., 1999). This process limits ease and the yield of *E. coli* expression, in particular for haloarchaeal proteins containing metallocofactors (Esclapez et al., 2006; Martínez-Espinosa, 2020).

Therefore, suitable haloarchaeon expression systems are often necessary. Successful homologous expression of haloarchaeal proteins has been reported in halophilic archaeal hosts, such as *Halobacterium salinarum* (Nomura and Harada, 1998; Kixmüller and Greie, 2012; Vauclare et al., 2020) and *Haloferax volcanii* (*Hfx. volcanii*) (Pohlschroder and Schulze, 2019; Haque et al., 2020). In particular, *Hfx. volcanii* has emerged as the microbe of choice for haloarchaeal genetics (Allers and Mevarech, 2005) and for developing systems for the successful overexpression and purification of halophilic proteins (Allers, 2010; Allers et al., 2010). A decade of research on haloarchaeon *Hfx. volcanii* enabled the development of host strains and plasmid vectors for overexpression of halophilic proteins (Allers, 2010; Allers et al., 2010; Pohlschroder and Schulze, 2019).

For both expression systems, *Hfx. volcanii* (Allers et al., 2010) and *E. coli* (Malash et al., 2020), new methods for the large-scale production of polyextremophilic proteins are currently being developed and refined. However, the tools available for the genetic manipulation of archaea are still scarce compared with those for bacteria, complicating the establishment of expression procedures. Moreover, since most enzymes contain metals or metallocofactors to obtain their catalytic functionality, establishing expression systems for haloarchaeal proteins containing metallocofactors in archaean hosts is a primary future objective (Martínez-Espinosa, 2020).

## Enzymes From the Red Sea and Their Potential Biotechnological Application

The few metagenomic studies conducted in the Red Sea revealed that the microbiota, and therefore the proteome, differ substantially from those found in other marine environments and are also markedly different between Red Sea brine pools (Thompson et al., 2013; Abdallah et al., 2014).

Ziko et al. (2019) revealed 2,751 specialized metabolism gene clusters in Atlantis II, Discovery and Kebrit brine pools while conducting a genome analysis with a focus on antibacterial and anticancer research. Sequenced metagenomes from Atlantis II and Discovery deep brine pools reveal marked differences between the two brine pools. Whereas the Atlantis II metagenome is inhabited predominately by bacteria, Discovery harbors mostly autotrophic archaea (Wang et al., 2011, 2013). A taxonomic analysis of sequenced environmental samples from the Red Sea brine pool sediments allowed us to categorize the existing microbial communities and to discover their roles in methane and sulfur cycling processes (Siam et al., 2012). A characterization of the microbial populations in different strata of the vertical brine pool profile highlighted the effects of salinity and temperature on shaping these microbial communities (Bougouffa et al., 2013). A gene cluster analysis from the brine-seawater interface demonstrated the diversity of methanotrophs in Atlantis II, Discovery and Kebrit (Abdallah et al., 2014). Metabolome studies of extremophilic microbiota in the Red Sea brine pools in Atlantis II, Kebrit and Discovery deeps predicted several new biomedical compounds with the potential to become new drugs (Ziko et al., 2019).

To date, a total of twelve enzymes have been characterized experimentally from Atlantis II, Discovery, and Kebrit (Table 1). Most of these extremozymes belong to three classes of enzymes, namely, oxidoreductases, transferases, and hydrolases.

**TABLE 1:** Extremozymes from the Red Sea brine pools.

Entry	Enzyme	NCBI GenBank	EC Number	Location	Characteristics	Thermostability	Halophilic	Metal tolerance	Expression system	References
1	Alcohol dehydrogenase ADH/A1a	KXB02677	1.1.1.1	Atlantis II Deep	Thermostable: 70°C; Salt: 3 M NaCl, 4 M KCl; withstands organic solvents	Thermophilic	X		*Hfx. volcanii*	Akal et al., 2019
2	5,6-dihydroxy NADPH- bound alcohol dehydrogenase ADH/D1	KXA95890.1	1.1.1.2	Discovery Deep	Thermostable: optimum 70°C; Salt: 2 M NaCl, 4 M KCl	Thermophilic	X		*E. coli* K12, *Hfx. volcanii* H1895	Grotzinger et al., 2018
3	Thioredoxin reductase ATII-TrxR		1.8.1.9	Atlantis II Deep	Thermostable: 60% activity at 70°C; Salt: up to 4 M NaCl; heavy metals tolerant	Thermophilic	X	X	*E. coli* BL21 (DE3)	Badiea et al., 2019
4	MerA mercuric reductase ATII-LCL	KF572479	1.16.1.1	Atlantis II Deep	Thermostable: 70% activity at 70°C	Thermophilic	X	X	*E. coli* BL21 (DE3)	Sayed et al., 2014; Maged et al., 2019
5	MerA mercuric reductase ATII-LCL-NH	MF363137	1.16.1.1	Atlantis II Deep	Thermostable: 81% activity at 60°C; non-halophilic	Thermophilic		X	*E. coli* BL21 (DE3)	Maged et al., 2019
6	K09H MerA mercuric reductase	KY421641	1.16.1.1	Kebrit Deep	Salt: 2 M NaCl		X		*E. coli* BL21 (DE3)	Ramadan et al., 2019
	K35NH MerA mercuric reductase	KY421666	1.16.1.1	Kebrit Deep	Non-halophilic				*E. coli* BL21 (DE3)	Ramadan et al., 2019
7	Brine pool-3 polymerase BR3 pol	KXB03331	2.7.7.7	Atlantis II Deep	Thermostable: 100% activity at 65°C; Salt: 0.5 M NaCl; tolerant to MgCl_2_ and MnCl_2_, ability to utilize Zn^2+^ ions	Thermophilic	X	X	*E. coli* BL21 (DE3) pLysS	Hamdan and Takahashi, 2015; Takahashi et al., 2018
8	3′-aminoglycoside phosphotransferase ATII-APH(3′)	KX377799	2.7.1.95	Atlantis II Deep	Thermostable: 40% activity at 65°C	Thermophilic			*E. coli* BL21 (DE3)	Elbehery A. H. et al., 2017
9	Esterase EstATII	KC958722.1	3.1.1	Atlantis II Deep	Thermostable: 77% activity at 75°C; Salt: up to 4.5 M NaCl; heavy metals tolerant	Thermophilic	X	X	*E. coli* BL21 (DE3)	Mohamed et al., 2013
10	Class A beta-lactamase ATII-ABL	KX377801	3.5.2.6	Atlantis II Deep	Thermostable: 43.3°C	Thermophilic			*E. coli* BL21 (DE3)	Elbehery A. H. et al., 2017
11	338 amino-acid nitrilase NitraS-ATII	KT354778	3.5.5.1	Atlantis II Deep	Thermostable: 60% activity at 70°C; heavy metals tolerant	thermophilic		X	*E. coli* BL21 (DE3)	Sonbol et al., 2016
12	γ-carbonic anhydrase CA_D	KXA95168.1	4.2.1.1	Discovery Deep	Halophilic	thermophilic	X		*Halobacterium* sp. NRC-1	Vogler et al., 2020

*The X represents characterization information provided in the corresponding publication.*

Oxidoreductases catalyze biological oxidation/reduction reactions. They perform their functions on different substrates, both organic and inorganic. These enzymes are applied in fields such as polymer synthesis, biodegradation of pollutants, development of biosensors and diagnostic tests (van den Burg, 2003; Selles Vidal et al., 2018; Atalah et al., 2019). Alcohol dehydrogenases (ADHs) are oxidoreductases used for the production of chiral compounds in the pharmaceutical and chemical industries due to their regio- and enantioselectivity (Zheng et al., 2017). To date, two ADHs have been discovered: (i) ADH/D1 (Grotzinger et al., 2018) from the Discovery Deep brine pool (2,141 m depth, 44.8°C, 26.2% salt, pH 6.4) and (ii) ADH/A1a (Akal et al., 2019) from the Atlantis II Deep brine pool (2,036 m depth, 63°C, 16.8% salt, pH 5.3). Both ADHs are thermostable, halophilic, withstand organic solvents, and accept primary long-chain and aromatic alcohols as substrates (Grotzinger et al., 2018; Akal et al., 2019). ADH/A1a oxidized a broad spectrum of alcohols. In the reduction reaction, cinnamaldehyde, cinnamyl-methyl-ketone, and raspberry ketone were exclusively reduced (Akal et al., 2019). Conversely, ADH/D1 showed a high specific activity toward cinnamyl alcohol (Grotzinger et al., 2018).

The current use of cinnamyl aldehyde and cinnamyl alcohol in the flavor and perfume industries expands the biotechnological potential of these enzymes beyond the production of precursors for pharma (Beutner and von Krogh, 1990; Youn et al., 2006).

Thioredoxin reductases (TrxRs) also belong to the oxidoreductase family and are involved in maintaining the redox environment of the cell by the reduction of thioredoxin (Mustacich and Powis, 2000; Saccoccia et al., 2014). Since TrxR activity is closely linked to cell growth and survival, it is a potential target for cancer therapy and novel antibiotics (Nguyen et al., 2006; Harbut et al., 2015). The common characteristic of TrxRs is the redox-active tetrapeptide motif containing a selenocysteine for catalytic activity (Lothrop et al., 2014). Thus, most drugs are based on selenocysteine inhibition (Becker et al., 2000; Saccoccia et al., 2014). However, according to one study, the halophilic and thermostable thioredoxin reductase ATII-TrxR from the Atlantis II Deep brine pool (2,200 m depth, 68°C, 26% salt, pH 5.3) lacks this selenocysteine (Badiea et al., 2019). This feature could potentially lead to a broad-spectrum drug that is not based on selenocysteine inhibition.

Another industrially important subclass is metal ion oxidoreductases, which increase the oxidation states of metals. Mercuric reductases (MerAs), for example, can attenuate the toxicity of mercury and are used in bioremediation (Selles Vidal et al., 2018). There are four known mercuric reductases isolated from the Red Sea brine pools that appear to have different properties in terms of thermostability, halophilicity, and metal tolerance. The first discovered mercuric reductase, ATII-LCL MerA, is from the lower convective layer of the Atlantis II Deep brine pool (2,000 m depth, 68°C, 26% salt, pH 5.3). ATII-LCL MerA is thermostable and halophilic and seems to be less affected by mercury inhibition than other MerAs (Sayed et al., 2014). More recently, two MerA isoforms, K09H and K35NH, from Kebrit Deep (1,549 m depth, 23.3°C, 26% salt, pH 5.5) were identified and characterized. Whereas K35NH MerA is strongly inhibited by high salt concentrations, K09H MerA shows the usual characteristics of halophilic proteins. Collectively, these works enhanced our understanding of salt adaptation and how environmental stressors shape the structure of orthologous enzymes while retaining their catalytic function. Given their stability and specificity, brine pool MerA enzymes are promising candidates for improved bioremediation and mercury detoxification, thereby mitigating the hazards of the mining industry (Sayed et al., 2014; Maged et al., 2019; Ramadan et al., 2019).

Generally, transferases do not play a major role in industrial processes, despite their catalytic function of transferring a non-hydrogen moiety between a pair of substrates (Singh et al., 2016; Paul et al., 2019). However, one transferase subgroup, DNA polymerases, is widely used for DNA manipulation, sequencing, labeling, mutagenesis, and other purposes (Ishino and Ishino, 2014). In particular, thermostable DNA polymerases are important for nucleic acid amplification techniques in molecular biology (Ishino and Ishino, 2014). Archaeal DNA polymerases are frequently used, and engineering chimeric archaeal DNA polymerases with increased processivity and fidelity is an emerging topic (Zhang et al., 2015). A recently discovered DNA polymerase from the Atlantis II Deep brine pool (unspecified depth, 55°C, 24% salt, pH not described), BR3 pol, has been shown to be active at relatively low temperatures of approximately 55°C. However, BR3 pol demonstrated unusual tolerance to high salt and metal ion concentrations, together with the unique ability to use Zn^2+^ as a cofactor (Takahashi et al., 2018). Takahashi et al. (2018) were able to engineer a chimeric DNA polymerase, combining BR3 Pol salt stability with the heat stability and performance of a *Thermococcus kodakarensis* DNA polymerase. The resulting halophilic chimera has been patented and is used for the development of new detection essays (Hamdan and Takahashi, 2015; Takahashi et al., 2018).

Due to the ongoing COVID-19 pandemic, there is a growing demand for rapid diagnostic tests, such as isothermal application methods (Zhao et al., 2015) like LAMP and/or RT-LAMP, to improve the efficiency and coverage of medical screening of infectious diseases (Ganguli et al., 2020; Obande and Banga Singh, 2020). In this situation, the robust and well-performing BR3 pol chimera is a commercially promising polymerase for isothermal applications.

Antibiotic resistance is increasingly threatening health care systems worldwide (Aslam et al., 2018; Morel et al., 2020). Tackling the emergence of multiresistant species requires (i) the development of new antibiotics and (ii) a better understanding of antibiotic resistance genes. Due to their location, the Red Sea brine pools are an interesting source for investigating the development of antibiotic resistance. Elbehery A. H. et al. (2017) found two novel antibiotic resistance enzymes from the Atlantis II Red Sea brine pools, a class A beta-lactamase, ATII-ABL, and the first ever reported thermostable 3′-aminoglycoside phosphotransferase, ATII-APH(3′). Class A beta-lactamases represent one of the major resistance mechanisms to fight β-lactam antibiotics which are the most widely used and effective antibiotics. To overcome this threat, novel and improved β-lactamase inhibitors have to be developed (Eiamphungporn et al., 2018). Aminoglycoside kinases, such as ATII-APH(3′), inactivate the antibiotic by reducing its affinity for the bacterial ribosome (Fong et al., 2005; Shi et al., 2013). Both antibiotic resistance genes may be used as thermophilic selection markers for thermophilic hosts. Furthermore, studies of phosphotransferases are required to obtain a better understanding of antibiotic resistance and to design inhibitors (Stogios et al., 2016; Terekhov et al., 2020).

The most widely studied class of enzymes is that of hydrolases, due to their catalytic promiscuity (Adler-Nissen, 1982; Selles Vidal et al., 2018). Hydrolases are used in organic biosynthesis, in the pulp and paper industry, in wastewater treatment, and for improving the digestibility of animal feed (Delgado-García et al., 2012; Flores-Gallegos et al., 2019). Esterases, a subclass of hydrolases, are utilized in textile manufacturing, flavor modifications in the food industry, oil biodegradation, synthesis of pharmaceuticals, and fine chemicals (Panda and Gowrishankar, 2005). Their lipolytic properties also make them prospects in biodiesel production (Yeoman et al., 2010). A recently discovered thermophilic halotolerant esterase from the lower convective layer of the Atlantis II Deep brine pool (2,000 m depth, 68.2°C, 26% salt, pH 5.3), EstATII, is a promising biocatalyst. EstATII shows high activity under a wide range of temperatures (30–80°C) and high salt concentrations (2–4.5 M NaCl). Moreover, the activity of EstATII is not affected by heavy metals, in contrast to other esterases (Mohamed et al., 2013). The substrate scope of EstATII is mainly restricted to short straight-chain alkyl carboxylic acids. Generally, the increased thermostability of esterases allows applications involving poorly soluble substrates (or products) for the synthesis of intermediates for the cosmetic industry (Ravot et al., 2004).

Another subclass of hydrolases is nitrilases (NitraS), which are used in the synthesis of pharmaceuticals (or their precursors), pesticides and bioremediation of cyanide (Thuku et al., 2009; Gong et al., 2012). Nitrilases have been used as green catalysts for the production of high value-added products due to their high selectivity and lack of toxic byproduct formation (Shen et al., 2021). Nitrilase NitraS-ATII was discovered in the lower convective layer of the Atlantis II deep brine pool (2,000 m depth, 68°C, 26% salt, pH 5.3). Compared to other nitrilases, NitraS-ATII is more stable at higher temperatures and maintains its activity in the presence of several metals. There are currently no thermostable nitrilases available on the commercial enzyme market, making the Red Sea a promising source for these enzymes (Sonbol et al., 2016; Atalah et al., 2019).

Lyases catalyze the cleavage of various chemical bonds, do not require cofactor recycling, and show high (stereo)specificity. Thus, lyases are a biocatalytic attractive enzyme class that are already used in several commercial processes (van der Werf et al., 1994). Carbonic anhydrases (CAs), a subclass of lyases, are common metalloenzymes in all domains of life. CAs catalyze the reversible hydration of carbon dioxide to bicarbonate and are used in several industrial applications (Frost and McKenna, 2013). Currently, carbonic anhydrases are being investigated for several cascade reactions involving the synthesis of small organic molecules (Yoshimoto and Walde, 2018), biofuel production (Bajracharya et al., 2017), and CO_2_ capture (Frost and McKenna, 2013). Because the increase in atmospheric CO_2_ due to fossil fuel combustion contributes to global warming and ocean acidification, measures such as CO_2_ capture and sequestration are explored. Several studies have shown that immobilized carbonic anhydrases can accelerate the rates of absorption of CO_2_ in the liquid phase (Savile and Lalonde, 2011; Supuran and Capasso, 2018; Yoshimoto and Walde, 2018). However, such processes require enzymes that are active under harsh conditions. γ-Carbonic anhydrase, CA_D, from the polyextreme Red Sea brine pool Discovery Deep (2,141 m depth, 44.8°C, 26.2% salt, pH not determined) is a potential candidate to overcome this limitation (Vogler et al., 2020). CA_D shows all characteristics of a halophilic protein and is active under elevated temperatures. Furthermore, a structure-driven mutagenesis study showed that the activity of the wild type can be increased by 17-fold (Vogler et al., 2020).

## Molecular Basis for Polyextremophilicity

In the last decade, the combination of structural biology and biochemistry has clarified the structure-function relationships of many biocatalysts (Pegg et al., 2006; Liang et al., 2019; Sriwaiyaphram et al., 2020). Studying (poly)extremophilic enzymes at the molecular level helps to understand the mechanisms of adaptation to extreme environments and facilitates the development of rational enzyme engineering strategies to turn mesophilic proteins into more stable versions (Angelaccio, 2013; Karan et al., 2020).

**FIGURE 2:**
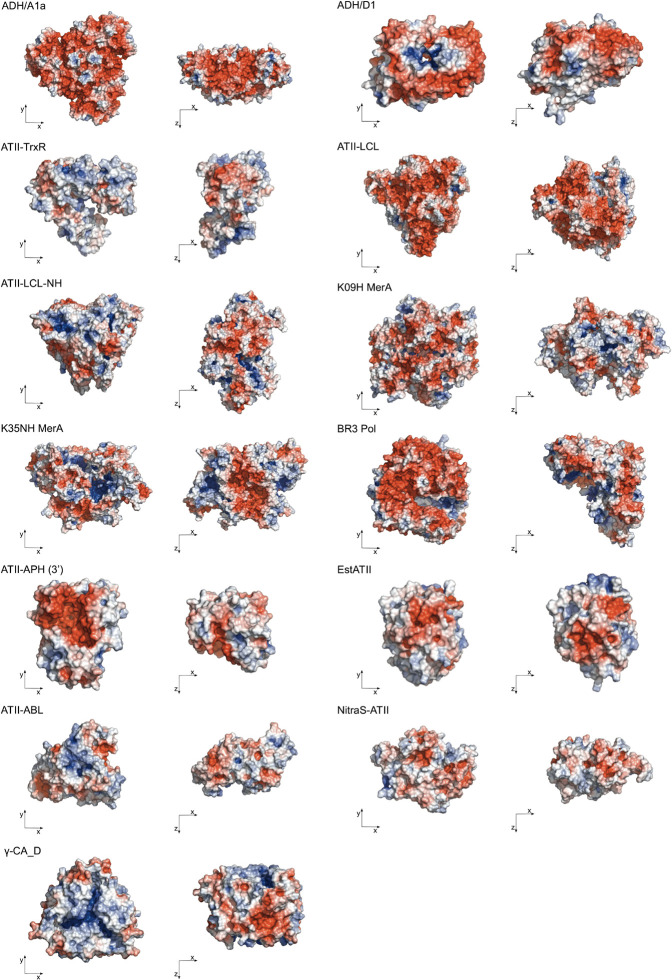
Surface representation of the extremozymes from the Red Sea brine pools. Surface colors indicate positive and negative electrostatic potentials contoured from 50 kT/e (blue) to –50 kT/e (red). The Phyre2 tool (Kelley et al., 2015) was used for homology modeling, with an average 100% confidence level at >90% accuracy. Visualized by PyMOL Molecular Graphics System, Version 2.4.2, Schrödinger, LLC.

The 3D structures of extremozymes are very valuable for rationalizing enzyme engineering strategies. Thermophilic and halophilic proteins tend to be more stable than mesophilic proteins and, therefore, may crystallize even when the mesophilic counterpart fails to do so (Jenney and Adams, 2008). Nonetheless, to date, only two Red Sea brine pool enzymes have been crystallized, namely, 5,6-dihydroxy NADPH-bound ADH/D1 alcohol dehydrogenase (PDB code: 5YVM) (Grotzinger et al., 2018) and γ-carbonic anhydrase CA_D (PDB code: 6SC4) (Vogler et al., 2020). Consequently, most structural studies of Red Sea enzymes still rely on structure-based homology models (Figure 2).

These models reveal that most Red Sea brine pool protein surfaces consist largely of negative charges, which enables higher salt tolerance by coordinating a water shell around the protein structure. The homotrimers ADH/A1a and γ-CA_D and the homodimers ADH/D1 and MerA exhibit positive electrostatic potential cores, which function as multimerization contacts (Figure 2). The only exceptions are EstATII and ATII-ABL, which show a higher positive electrostatic potential distribution on the surface. In addition to structural analyses, the physiological characteristics of extremozymes can also help to build rationale (Table 2).

**TABLE 2:** Physiological characteristics of extremozymes from the Red Sea brine pools.

Entry	Enzyme	Theoretical IEP	Negative charges	Positive charges	Grand average of hydropathicity (GRAVY)	Salt bridges	Aliphatic index
1	Alcohol dehydrogenase ADH/A1a	5.01	56	37	–0.254	119	89.25
2	5,6-dihydroxy NADPH-bound alcohol dehydrogenase ADH/D1	4.88	65	41	–0.279	20	85.42
3	Thioredoxin reductase ATII-TrxR	6.96	56	55	0.127	135	99.58
4	MerA mercuric reductase ATII-LCL	4.66	91	51	–0.185	227	90.64
5	MerA mercuric reductase ATII-LCL-NH	5.60	60	48	0.088	155	95.06
6	K09H MerA mercuric reductase	5.77	60	50	0.073	168	95.40
	K35NH MerA mercuric reductase	5.60	60	48	0.101	177	96.10
7	Brine pool-3 polymerase BR3 pol	5.16	148	117	–0.521	315	87.30
8	3′-aminoglycoside phosphotransferase ATII-APH(3′)	4.65	39	25	–0.028	134	87.79
9	Esterase EstATII	7.11	27	27	0.172	114	105.76
10	Class A beta-lactamase ATII-ABL	7.70	40	41	0.017	119	96.17
11	338 amino-acid nitrilase NitraS-ATII	6.24	38	32	–0.172	227	87.16
12	γ-carbonic anhydrase CA_D	5.92	29	24	–0.292	83	99.07

*The theoretical pI, negative charges, positive charges, grand average of hydropathicity (GRAVY) and aliphatic index were calculated by the Expasy server (Gasteiger et al., 2005). The number of salt bridges was calculated by ESBRI (Costantini et al., 2008).*

Collectively, these studies indicate that the structure-function relationship for haloadaptation is mainly achieved by an amino acid composition with greatly enriched negatively charged residues and minimal positively charged amino acids, especially lysines (Table 2). This is also reflected in the theoretical isoelectric point (IEP) and the grand average of hydropathy (GRAVY) (Kyte and Doolittle, 1982) values, indicating hydrophobic regions typical for marine halophilic proteins (Paul et al., 2008; Harding et al., 2016). Furthermore, weakly favored protein-ion interactions stabilize the folded state of halophilic proteins (Siglioccolo et al., 2011; Sinha and Khare, 2014; Ortega et al., 2015).

Similarly, thermophilicity is not accomplished by a single mechanism but by combining several stabilizing factors to maintain structure and function at high temperatures. Thermophilic proteins show a more rigid structure primarily manifested by an increased number of salt bridges (Table 2) and by side chain-side chain hydrogen bonds (Kumar et al., 2000; Razvi and Scholtz, 2006; Sawle and Ghosh, 2011). Moreover, the aliphatic index, which is an indirect thermostability index, is significantly higher for thermophilic proteins than mesophilic proteins (Table 2) (Ikai, 1980; Guruprasad et al., 1990; Devi et al., 2013).

These observations suggest that the structural adaptations of the proteins from the Red Sea brine pool microorganisms are similar to those of others in extreme environments. The adaptation to high temperature and high salt concentration is facilitated by a compact hydrophobic core and increased salt bridge interactions that help to maintain the structural integrity. A high number of negatively charged random coils contribute to dynamic flexibility. Three extremozymes from the Red Sea brine pools have already been used to test the hypothesis of structural adaptation or for engineering approaches. In a first mutagenesis study, the mercuric reductase ATII-LCL MerA was transformed into a non-halophilic enzyme with reduced thermostability while maintaining the kinetic activity of the wild-type enzyme. In line with the importance of acidic protein surfaces in conferring salt tolerance, the MerA mutant library used was based on only three defined regions within the expected dimerization domain, in which mostly aspartic acids were replaced by alanines. Remarkably, the knowledge gained was then used to design a mutant with increased stability (Maged et al., 2019).

Second, specific regions of the brine pool DNA polymerase BR3 Pol (Hamdan and Takahashi, 2015; Takahashi et al., 2018) (exonuclease, fingers, and thumb domains) were swapped with domains of a *T. kodakarensis* DNA polymerase, resulting in catalytically active chimeric DNA polymerases with higher salt stability. All swapped domains increased the salt stability compared to the wild type. However, none of the chimeric DNA polymerases reached the salt tolerance level of BR3 Pol, indicating that all domains contribute to halophilicity. Further investigations of the thermotolerance of the chimeric DNA polymerases showed that the increased salt tolerance comes with the price of instability at elevated temperatures, as most of the chimeric DNA polymerases lost their activity.

Last, engineering of γ-carbonic anhydrase CA_D (Vogler et al., 2020) selectively and specifically increased the activity of the enzyme. The authors enhanced the activity by substituting key residues in the active site with the corresponding residues from more active homologs. Thus, the γ-carbonic anhydrase CA_D backbone worked as a scaffold, preserving the halophilicity and thermophilicity while increasing the activity.

These mutagenesis and chimerization studies, along with studies conducted on extremozymes from other sources, enhance our understanding of structural adaptations to polyextremophily and contribute to engineering approaches to introduce stability characteristics to mesophilic proteins.

## Conclusion and Future Perspectives on Red Sea Brine Pool Proteomic Research

Over the last decades, extremozymes from deep-sea extremophiles have emerged as a promising source for novel and robust enzyme variants that are much needed for use in industrial settings. Their evolutionary adaptations to harsh conditions make them promising candidates for more robust biocatalysts. Consequently, these novel extremozymes are expected to enable biocatalytic process engineering that is more efficient, sustainable and environmentally friendly than current chemical processes.

The few metagenomic studies on the Red Sea brine pools provide only a first glimpse into the enormous biodiversity of microorganisms and their potential purposes. Completing only the microbial picture of the Red Sea brine pools, as a selected niche, is already a challenging task that can only be achieved by systematic sampling and NGS frameworks combined with a suitable metagenomic toolset. However, even this framework cannot capture the dynamics of this marine ecosystem that are continuously changing and will only provide a snapshot of the microbial profiles.

Several databases are known for holding metagenomic data isolated from Red Sea brine pools. However, only a few are regularly updated. Thus, metagenomic data are already available, yet they are not compiled in one place. This makes a systematic screening of Red Sea (extremo)enzymes almost impossible. A dedicated metagenomic annotation database would accelerate and simplify the search for novel robust industrially applicable enzyme variants from Red Sea brine pools.

Metagenomic annotated data are a good starting point for selecting potential industrially applicable enzyme variants. However, suitable genetic screening techniques for direct gene or gene cluster screening in the Archaea expression systems are still missing. This severely limits the application of systematic high-throughput screening to confirm the selected candidates.

Methodological limitations constrain not only the discovery of novel biocatalysts, but also the fine-tuning for industrial applications. An elegant way to overcome this limitation is to use extremophiles directly as production microorganisms in industrial applications, but this presents several difficulties. Too little is known about potential toxic side products, behavior in bioreactor systems, or suitable large-scale purification. Developing new culture and molecular tools, scale-up procedures, and new methods for protein engineering will facilitate the potential applications of extremozymes in industry.

Recently, a new brine pool within the Red Sea has been discovered. Afifi is located on the eastern shelf of the southern Red Sea and is described as highly saline (228 g/L), cold (23.3°C), and anoxic; it is the shallowest brine basin yet reported in the Red Sea, with a depth range of 353–400 m (Duarte et al., 2020). This recent discovery highlights how unexplored the Red Sea is and the growing potential of Red Sea extremozymes from brine pools.

The progression of modern molecular methods in combination with deep-sea sampling approaches will allow extremozymes to significantly impact a wide range of industries in the future.

## Author Contributions

All authors listed have made a substantial, direct and intellectual contribution to the work, and approved it for publication.

## Conflict of Interest

The authors declare that the research was conducted in the absence of any commercial or financial relationships that could be construed as a potential conflict of interest.

## Publisher’s Note

All claims expressed in this article are solely those of the authors and do not necessarily represent those of their affiliated organizations, or those of the publisher, the editors and the reviewers. Any product that may be evaluated in this article, or claim that may be made by its manufacturer, is not guaranteed or endorsed by the publisher.

## Footnotes

1
http://www.cbrc.kaust.edu.sa/indigo
